# Study of female pelvic floor muscle in overactive bladder based on MRI 3D reconstruction

**DOI:** 10.1186/s12894-022-01090-9

**Published:** 2022-08-27

**Authors:** Yangyun Wang, Jie Yao, Na Chen, Jingjing Liu, Guowei Shi, Yi Wu

**Affiliations:** 1 Department of Urology, Shanghai Fifth People’s Hospital, Fudan Univeristy, Shanghai, 200240 China; 2 Department of Digital Medicine, College of Biomedical Engineering and Medical Imaging, Army Medical University (Third Military Medical University), Chongqing, 400038 China

**Keywords:** Overactive bladder (OAB), Pelvic floor muscle, Pelvic organ prolapse (POP), Three-dimensional reconstruction (3D reconstruction), MRI

## Abstract

**Background:**

This study examined the three-dimensional (3D) morphological changes of the urination and urinary continence anatomical structures in overactive bladder (OAB) patients, to offer a morphological data for OAB diagnosis and treatment.

**Methods:**

Eleven OAB patients, 9 healthy females and 22 pelvic organ prolapse (POP) patients were enrolled and underwent MRI scans. The anatomical components of urination (bladder detrusor) and the urinary continence (main part of the urethral sphincter, compressor urethrae, and levator ani muscle (LAM) were 3D reconstructed and measured with Amira software. We also analyze the relativity between pelvic floor muscle’s morphological parameters among the volunteers, OAB and POP group.

**Results:**

Through 3D reconstruction, increased thickness and volume of the bladder detrusor were found in the OAB patients compared with volunteers (3.1 ± 0.7 mm vs. 1.9 ± 0.3 mm; *P* = 0.000 and 50,632.0 ± 19,724.7 mm^3^ vs. 23,386.6 ± 7826.3 mm^3^; *P* = 0.001). The volume of LAM showed no significant difference between the OAB patients and volunteers (27,089.4 ± 5015.0 mm^3^ vs. 27,294.4 ± 4461.4 mm^3^; *P* = 0.924); whereas, LAM’s volume of the POP patients was significantly larger than that of the volunteers (34,130.6 ± 7968.3 mm^3^ vs. 27,294.4 ± 4461.4 mm^3^; *P* = 0.023). The thickness and volume of the main part of urethral sphincter were significantly lower in the OAB patients compared with volunteers (2.2 ± 0.5 mm vs. 2.7 ± 0.3 mm; *P* = 0.018 and 2558.6 ± 703.2 mm^3^ vs. 23,267.3 ± 681.9 mm^3^; *P* = 0.035). The volume of the compressor urethrae was significantly lower in the OAB patients than that in the volunteers (630.3 ± 301.2 mm^3^ vs. 866.1 ± 514.2 mm^3^; *P* = 0.247).

**Conclusions:**

In OAB patients, the bladder detrusor has long-term tension and contraction, which thickened muscle and increased volume, and aggravate urination. The compressor urethral and main part of urethral sphincter are weaker and the anterior part of LAM hiatus is relaxed, easily resulting in leakage of urine and ultimately incontinence. The MRI 3D reconstruction and measurement can help to evaluate pelvic floor urination and continence function, and accurately diagnose.

## Introduction

Overactive bladder (OAB) is a common clinical syndrome of the urinary system, characterized by symptoms such as urinary urgency, frequent micturition, and nocturia. The OAB easily leads to urge urinary incontinence (UUI), which deeply impacts the female quality of life. An incidence of 7.7% to 31.3% for OAB in women has been estimated in an epidemiological survey involving 19,000 adult women older than 18 in the United States, showing an age-based linear increase. It is currently a chronic disease more frequent than diabetes and peptic ulcers. Patients with a higher obesity index and body mass index (BMI) may develop severe OAB symptoms and often complicated with UUI.

OAB syndrome is often manifested with bladder hyperactivity, enhanced urination force and frequent contraction of the bladder detrusor, and problems in pelvic floor urinary continence (UC) anatomical elements, such as the urethral sphincter complex (USC) and levator ani muscle (LAM) weakness.

Christian et al. and Wu et al. conducted a digital anatomical study of the pelvic floor UC anatomical elements, including the LAM and compressor urethral, based on histological section images and thin-layer high-precision anatomical images. Their study illustrated that the female urethral elements consisted of the LAM, the main part of the urethral sphincter, the compressor urethral, and the urethrovaginal sphincter of the USC. Meanwhile, LAM and compressor urethral were regarded as the main UC anatomical elements. The contracted LAM compresses the rectum to press the urethra anterior-superiorly, while the contracted semicircular compressor urethral presses the urethra backward. Their contraction causes bending and resultant closure of the middle urethra for UC [1, 2].

For this reason, we speculated excessive hypertrophy of the urination anatomical element detrusor or weakened UC anatomical elements (USC and LAM) in OAB patients. Additionally, we surmised whether three-dimensional (3D) anatomical model construction of OAB patients and measurements of length, thickness, and volume can serve as an excellent quantitative evaluation tool for disease diagnosis and prognosis, contributing to OAB severity evaluation.

A non-invasive transvaginal color Doppler ultrasonography is frequently applied for OAB diagnosis. However, only two-dimensional tomographic images can be obtained through transvaginal ultrasonography, which is difficult to fully present the 3D morphological structures of the pelvic floor and insufficient for OAB diagnosis, preoperative management, and prognostic evaluation. Magnetic resonance imaging (MRI) is a continuous thin-slice high-precision imaging technique for clear identification, segmentation, and 3D reconstruction of muscles as well as measurement of muscle volume and intermuscular fat ratio. Many studies have reported correlations of muscle and intermuscular fat volumes with their function and disease diagnosis [3–5]. To assess the relevance of psoas muscle volume after menopause to acute osteoporosis or compression fractures, Huang et al. retrospectively collected spinal MRI images from postmenopausal women and performed 3D reconstruction and quantification analyses, suggesting a positive correlation of acute compression fractures with the muscle volume/fat volume ratio [5]. Additionally, de Figueiredo Melke et al. evaluated the function of the lateral pterygoid muscle (LPM) by calculating the muscle volume through MRI 3D reconstruction [6].

Therefore, we selected the MRI images of OAB patients, normal females, and patients with pelvic organ prolapse (POP), segmented the pelvic floor structures (LAM, USC, and bladder detrusor), followed by 3D reconstruction and measurements of length, thickness, and volume, to determine their differences in 3D morphological parameters of urination and UC anatomical elements, thus providing the morphological basis and reference for OAB diagnosis and treatment.

## Materials and methods

### Clinical data

The MRI images were selected from 11 female patients with OAB in Shanghai 5th Hospital, 9 healthy female volunteers, and 22 female patients with grade 2–4 POP in Southwest hospital from August 2018 to December 2020. The OAB patients were aged between 24 and 69 years, with a mean age of 42 ± 15.5 years; the healthy volunteers were aged between 23 and 70 years, with a mean age of 35.2 ± 13.9 years; the POP patients were aged between 24 and 74 years, with a mean age of 56.4 ± 13.3 years. The detailed basic data of the normal, OAB and POP group can be shown in Table 1.

**Table 1:** Basic characteristics of the OAB, POP group and normal group

	OAB group (n = 11)	Normal group (n = 9)	POP group (n = 22)
Age (years)	42 ± 15.5	35.2 ± 13.9	56.4 ± 13.3
BMI (kg/m^2^)	25.3 ± 0.6	21.7 ± 1.6	24.4 ± 2.6
Pregnancies	1.7 ± 0.6	0.6 ± 0.7	3.6 ± 1.4
Vaginal deliveries	1.4 ± 0.5	0.3 ± 0.5	2.2 ± 0.7
Abortions	0.4 ± 0.5	0.4 ± 0.7	1.4 ± 1.4

Values are given as mean ± standard deviation (SD) or number (percentage), unless indicated otherwise. Body mass index (BMI) is calculated as weight in kilograms divided by the square of height in meters. OAB, overactive bladder; POP, pelvic organ prolapse

### Inclusion and exclusion criteria

Inclusion criteria: (1) married female; (2) patients who met the diagnostic criteria for OAB (experimental group); (3) individuals with normal physical examination indicators (normal group); (4) patients who met the diagnostic criteria for POP (POP group); (5) patients with complete clinical data; (6) patients who voluntarily signed the informed consent.

Exclusion criteria: (1) patients with examination contraindications; (2) male patients; (3) patients complicated with other serious urinary system diseases, cardiovascular and cerebrovascular diseases, cardiopulmonary dysfunction, hepatic and renal insufficiency or malignant tumors; (4) patients complicated with pelvic organ dysfunction including pelvic organ prolapse and stress urinary incontinence, and other diseases that affect urination function such as lumbosacral trauma or history of surgery (OAB group); (5) patients complicated with mental illness, disturbance of consciousness, hearing disorders or language impairment; (6) patients complicated with bladder outlet obstruction (BOO); (7) individuals who did not receive anticholinergic drug therapy within 2 weeks of the examination; (8) individuals who were drop-out, transferred to another hospital, died or lost contact during follow-up; (9) incompliance to treatment or examination.

### MRI examination

All patients and volunteers underwent MRI. The scan parameters for OAB patients were set as follows: Repetition Time 3.58 s; Echo Time 1.35 s; Flip Angle 12.00; Pixel Bandwidth 445; Rows 250; Columns 320; Pixel Spacing 1.06\1.06; Slice Thickness 3 mm; Spacing Between Slices 3.00 mm. The scan range was from the upper border of the bladder to the lower border of the perineum.

Scan parameters for volunteers and POP patients were set as follows: Repetition Time 8610 ms; Echo Time 11 ms; Flip Angle 170; Pixel Bandwidth 319; Rows 320; Columns 320; Pixel Spacing 0.88\0.88; Slice Thickness 1 mm; Spacing Between Slices 1.2 mm. The scan range was from the upper border of the bladder to the lower border of the perineum.

### Image segmentation and 3D reconstruction

**Fig. 1:**
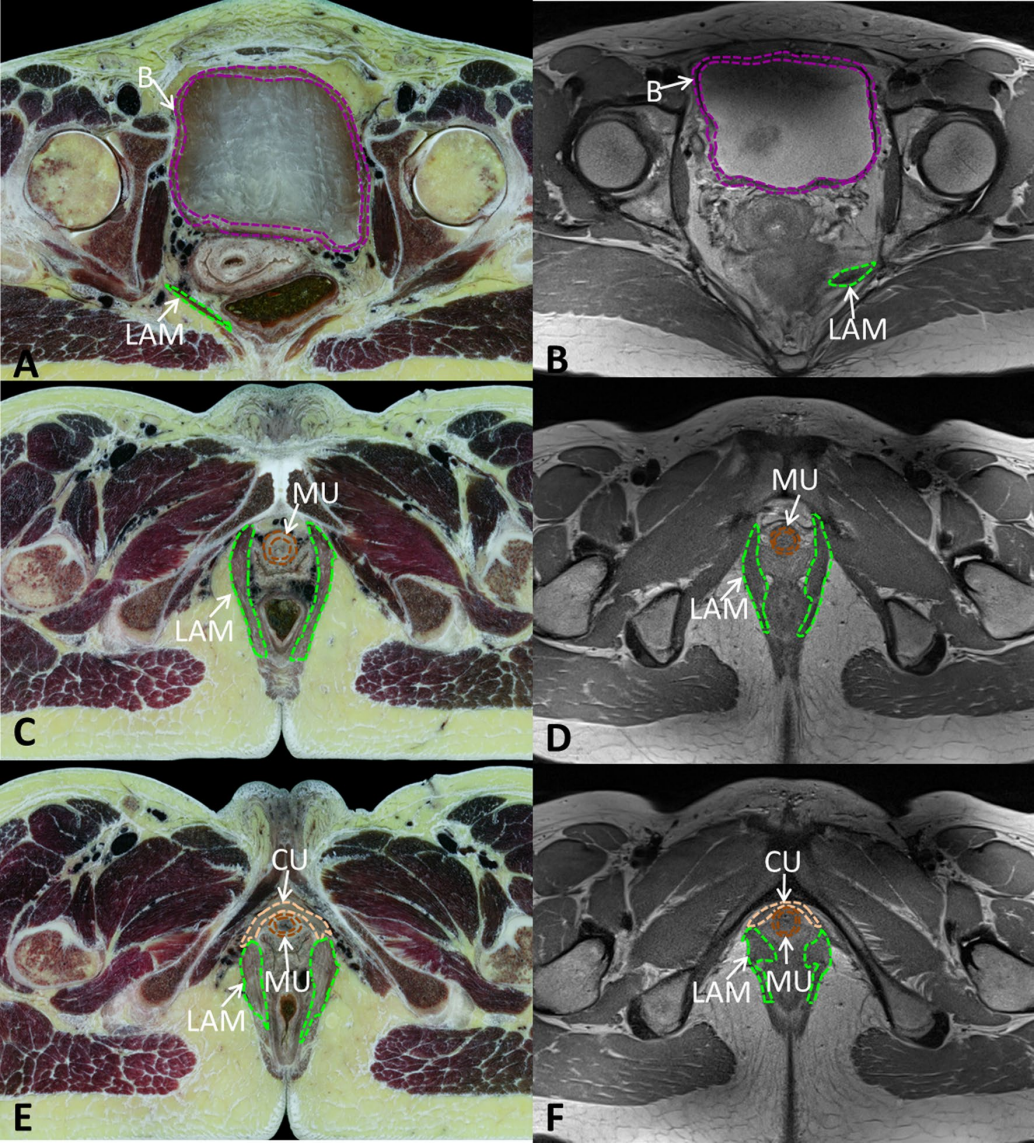
Comparison and segmentation between CVH and MRI pelvic floor. **A**, **B** Comparison and segmentation between CVH and MRI in bladder section; **C**, **D** Comparison and segmentation between CVH and MRI in superior urethra section; **E**, **F** Comparison and segmentation between CVH and MRI in inferior urethra section. CVH, Chinese Visible Human; LAM, levator ani muscle; B, bladder detrusor; MU, main part of urethral sphincter; CU, compressor urethrae

**Fig. 2:**
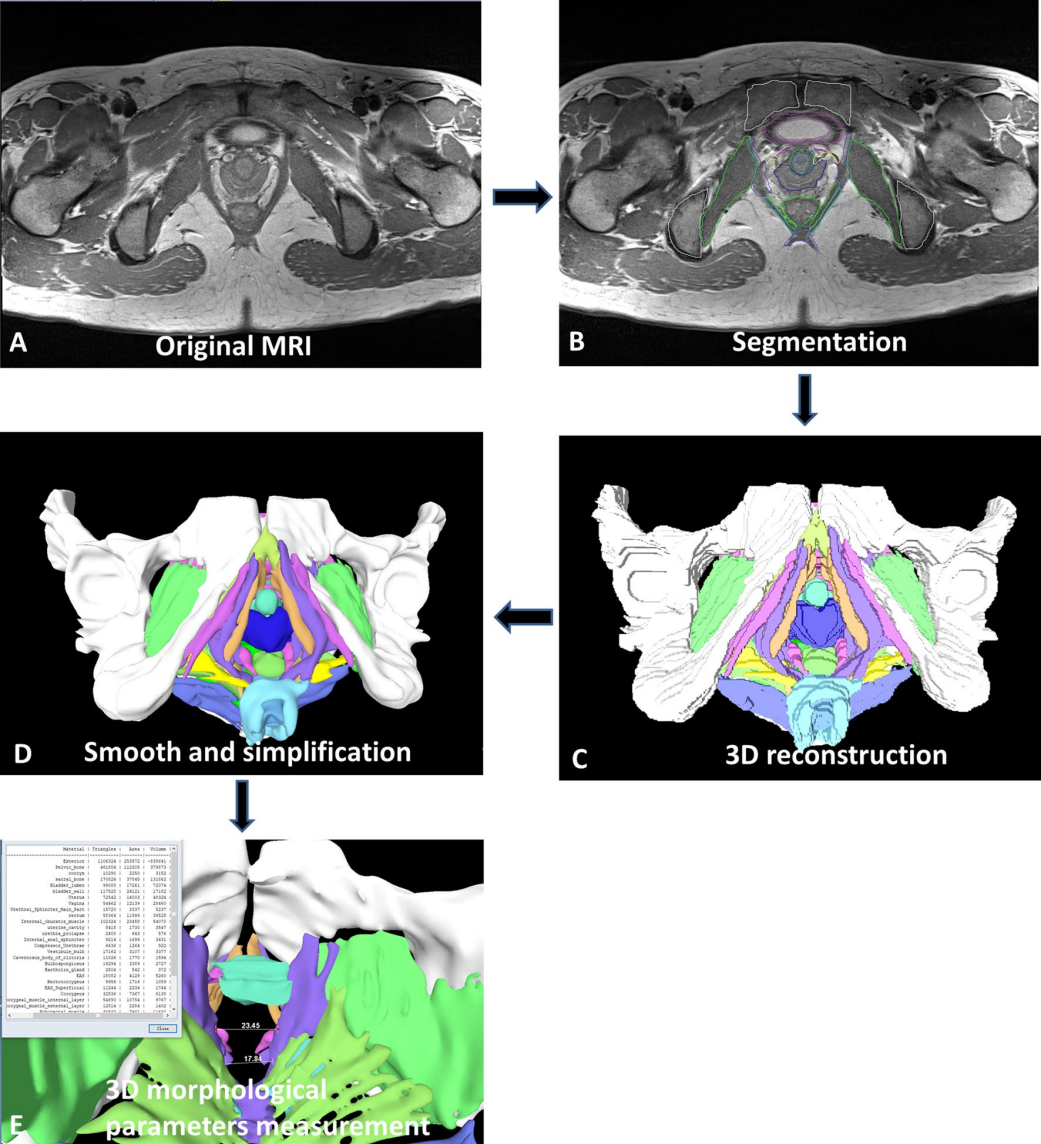
Image collection, segmentation, 3D reconstruction, model smoothing and simplification, and data measurement in normal, OAB and POP group. **A** Image collection; **B** Image segmentation; **C** 3D reconstruction; **D** model smoothing and simplification; **E** 3D measurement of the model

We imported MRI images with Amira software (Visage Imaging Corporation, Austrilia, http://www.amiravis.com, version 5.3.3) and compared the continuous thin-slice high-precision high-resolution tomographic anatomical images (Chinese Visible Human data set) [7, 8] with the previous segmentation results [2, 9, 10] (Fig. 1). We used Chinese Visible Human images to identify the pelvic floor structures in MRI, and we conducted data segmentation and 3D reconstruction of bladder detrusor, bladder lumen, main part of urethral sphincter, compressor urethral, and LAM. Accurate and smooth 3D digital models of individual structures were generated after smoothing and simplification using the Amira software (Fig. 2).

### 3D measurement

Using Amira software, we measured the thickness and volume of the main part of urethral sphincter, the diameter of the urethra, the thickness and volume of the bladder detrusor, the thickness, length, and volume of the compressor urethral, length of the anterior, middle, and posterior part of LAM hiatus, and the volume of the LAM.

### Statistical analysis

SPSS 19.0 software was utilized for data statistical analysis, and measurement data that conformed to normal distribution were expressed as mean ± standard deviation $({\overline{\text{x}}} \pm {\text{s}})$. Two independent samples *t*-test was adopted for comparison between groups, and Fisher's exact probability test was employed for comparison of enumeration data with a two-sided significance level set as α = 0.05. *p* < 0.05 was deemed statistically significant. The correlation between pelvic floor muscle morphological characters and basic characters in OAB patients were studied using the Spearman’s correlation analysis.

## Results

We completed 3D reconstruction and 3D morphological parameter measurements of 11 OAB patients, 9 volunteers, and 22 POP patients, and performed a retrospective analysis. Meanwhile, 3D digital models of the while pelvis of OAB, POP patients and volunteers were successfully created using Amira software. The measurement results are summarized in Tables 2 and 3.

**Table 2:** Comparison of pelvic floor muscle 3D characters between the OAB patients and the healthy volunteers

Pelvic floor muscle morphological characteristics	OAB group (n = 11)	Normal group (n = 9)	*P* value
Thickness of bladder detrusor (mm)	3.1 ± 0.7	1.9 ± 0.3	0.000
Volume of bladder detrusor (mm^3^)	50,632.0 ± 19,724.7	23,386.6 ± 7826.3	0.001
Thickness of main part of urethral sphincter (mm)	2.2 ± 0.5	2.7 ± 0.3	0.018
Volume of main part of urethral sphincter (mm^3^)	2558.6 ± 703.2	3267.3 ± 681.9	0.035
Diameter of the urethra (mm)	15.7 ± 1.6	15.7 ± 1.2	0.982
Thickness of compressor urethral (mm)	3.6 ± 0.7	3.7 ± 1.0	0.928
Length of compressor urethral (mm)	34.5 ± 6.6	38.2 ± 11.6	0.416
Volume of compressor urethral (mm^3^)	630.3 ± 301.2	866.1 ± 514.2	0.247
Length of the anterior part of LAM hiatus (mm)	41.4 ± 3.3	39.0 ± 5.5	0.279
Length of middle part of LAM hiatus (mm)	37.7 ± 5.0	36.7 ± 4.3	0.641
Length of posterior part of LAM hiatus (mm)	23.0 ± 6.0	21.2 ± 4.6	0.451
Volume of LAM (mm^3^)	27,089.4 ± 5015.0	27,294.4 ± 4461.4	0.924

OAB, overactive bladder; LAM, levator ani muscle

**Table 3:** Comparison of pelvic floor muscle 3D characters between the POP patients and the normal volunteers

Levator ani muscle morphological characteristics	POP group (n = 22)	Normal group (n = 9)	*P* value
Length of the anterior part of LAM hiatus (mm)	42.9 ± 7.3	39.0 ± 5.5	0.166
Length of middle part of LAM hiatus (mm)	43.6 ± 7.1	36.7 ± 4.3	0.010
Length of posterior part of LAM hiatus (mm)	27.0 ± 3.9	21.2 ± 4.6	0.001
Volume of LAM (mm^3^)	34,130.6 ± 7968.3	27,294.4 ± 4461.4	0.023

OAB, overactive bladder; POP, pelvic organ prolapse; LAM, levator ani muscle

### Construction of 3D digital models of OAB patients, volunteers, and POP patients

**Fig. 3:**
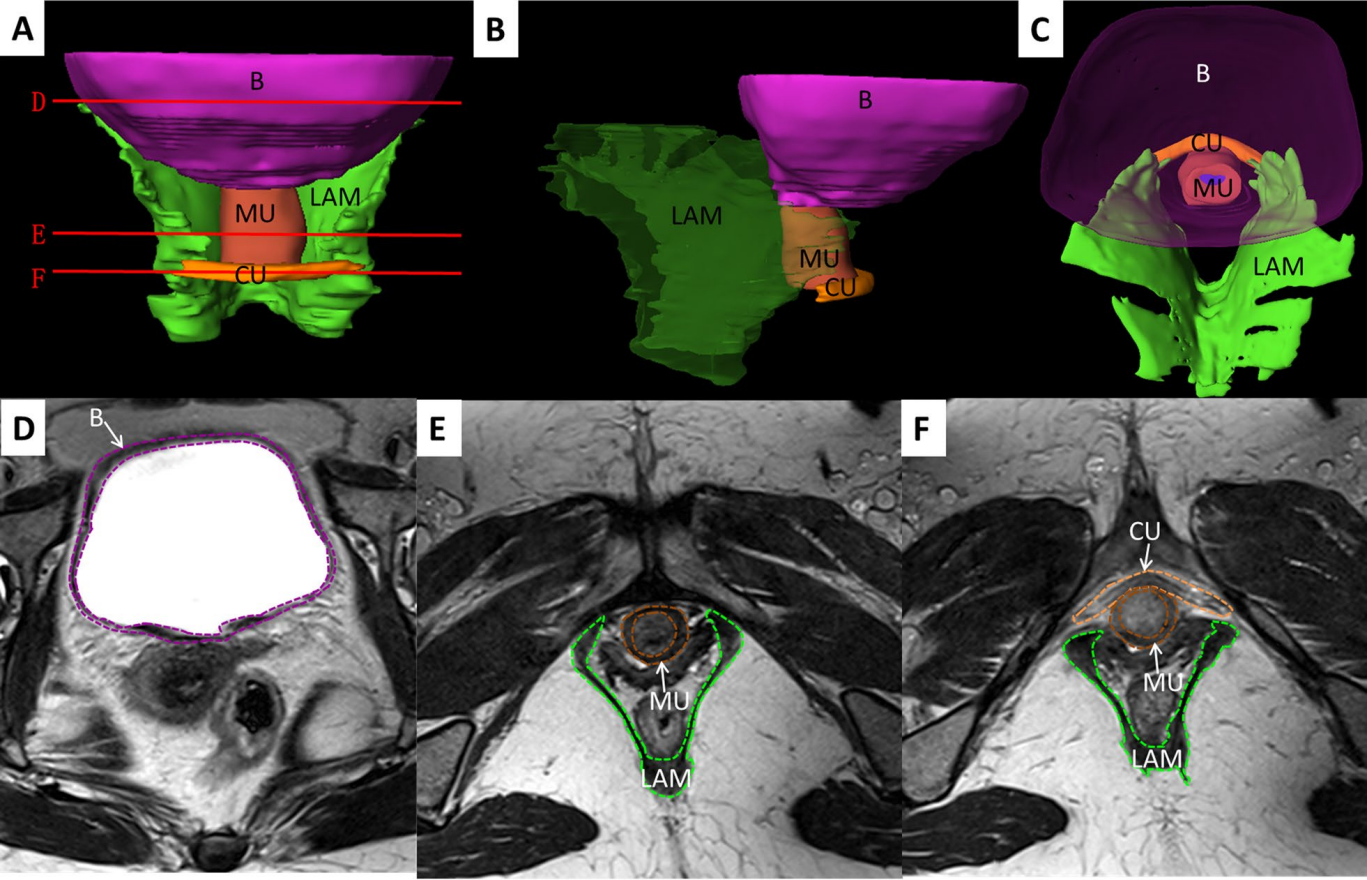
3D models and transverse MRI images of bladder detrusor and urinary continence components of OAB patients. **A** Anterior view; **B** left view; **C** superior view; **D** bladder section; **E** superior urethra section; **F** inferior urethra section. LAM, levator ani muscle; B, bladder detrusor; MU, main part of urethral sphincter; CU, compressor urethrae

**Fig. 4:**
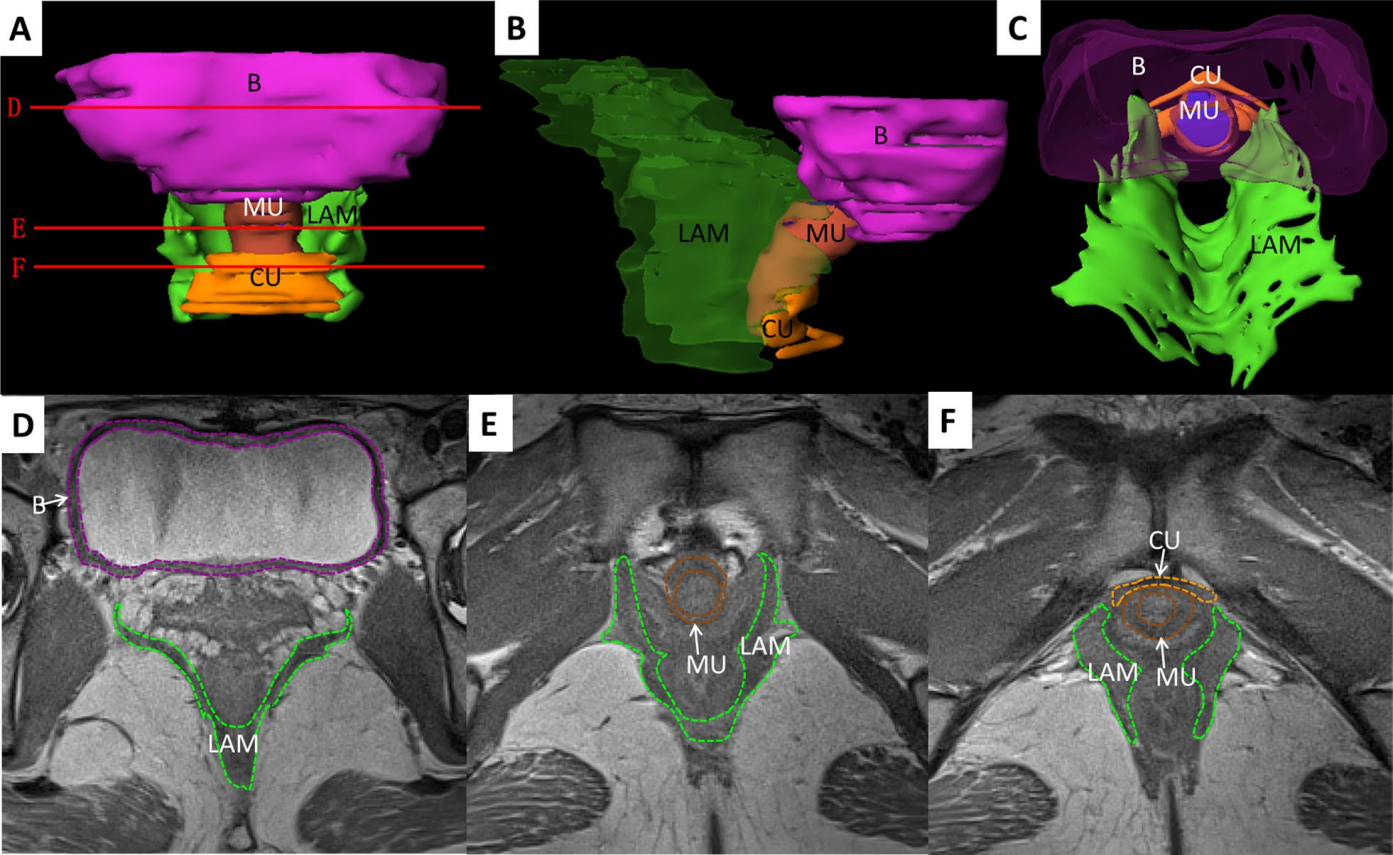
3D models and transverse MRI images of bladder detrusor and urinary continence components of healthy volunteers. **A** Anterior view; **B** Left view; **C** Superior view; **D** bladder section; **E** Superior urethra section; **F** Inferior urethra section. LAM, levator ani muscle; B, bladder detrusor; MU, main part of urethral sphincter; CU, compressor urethrae

We constructed 3D digital models of OAB patients, healthy volunteers, and POP patients, and found no significant 3D topological changes of the pelvic floor muscles in OAB patients when compared with the volunteers. However, the volume of bladder muscles in OAB patients (50,632.0 ± 19,724.7 mm^3^) was notably larger than that of healthy volunteers (23,386.6 ± 7826.3 mm^3^). We noticed significantly increased thickness of bladder detrusor in OAB patients (3.1 ± 0.7 mm) as compared to healthy volunteers (1.9 ± 0.3 mm). However, the volume and thicknesses of the main part of urethral sphincter in OAB patients (2558.6 ± 703.2 mm^3^ and 2.2 ± 0.5 mm) were significantly smaller than those of healthy volunteers (3267.3 ± 681.9 mm^3^ and 2.7 ± 0.3 mm). No significantly smaller volume, thickness, and length of the compressor urethral were observed in patients with OAB (630.3 ± 301.2 mm^3^, 3.6 ± 0.7 mm, and 34.5 ± 6.6 mm) relative to those of healthy volunteers (866.1 ± 514.2 mm^3^, 3.7 ± 1.0 mm, 38.2 ± 11.6 mm) (Figs. 3, 4).

The LAM volume showed almost no difference between the OAB patients and healthy volunteers. However, the widths of the anterior, middle, and posterior part of LAM hiatus (41.4 ± 3.3 mm, 37.7 ± 5.0 mm, and 23.0 ± 6.0 mm) were larger than those of the healthy volunteers (39.0 ± 5.5 mm, 36.7 ± 4.3 mm, and 21.2 ± 4.6) without statistical significance, among which the width of the anterior part of LAM hiatus showed the largest difference (about 2.4 mm). Almost no difference was found in the diameter of the urethra (Table 2) (Figs. 3, 4).

**Fig. 5:**
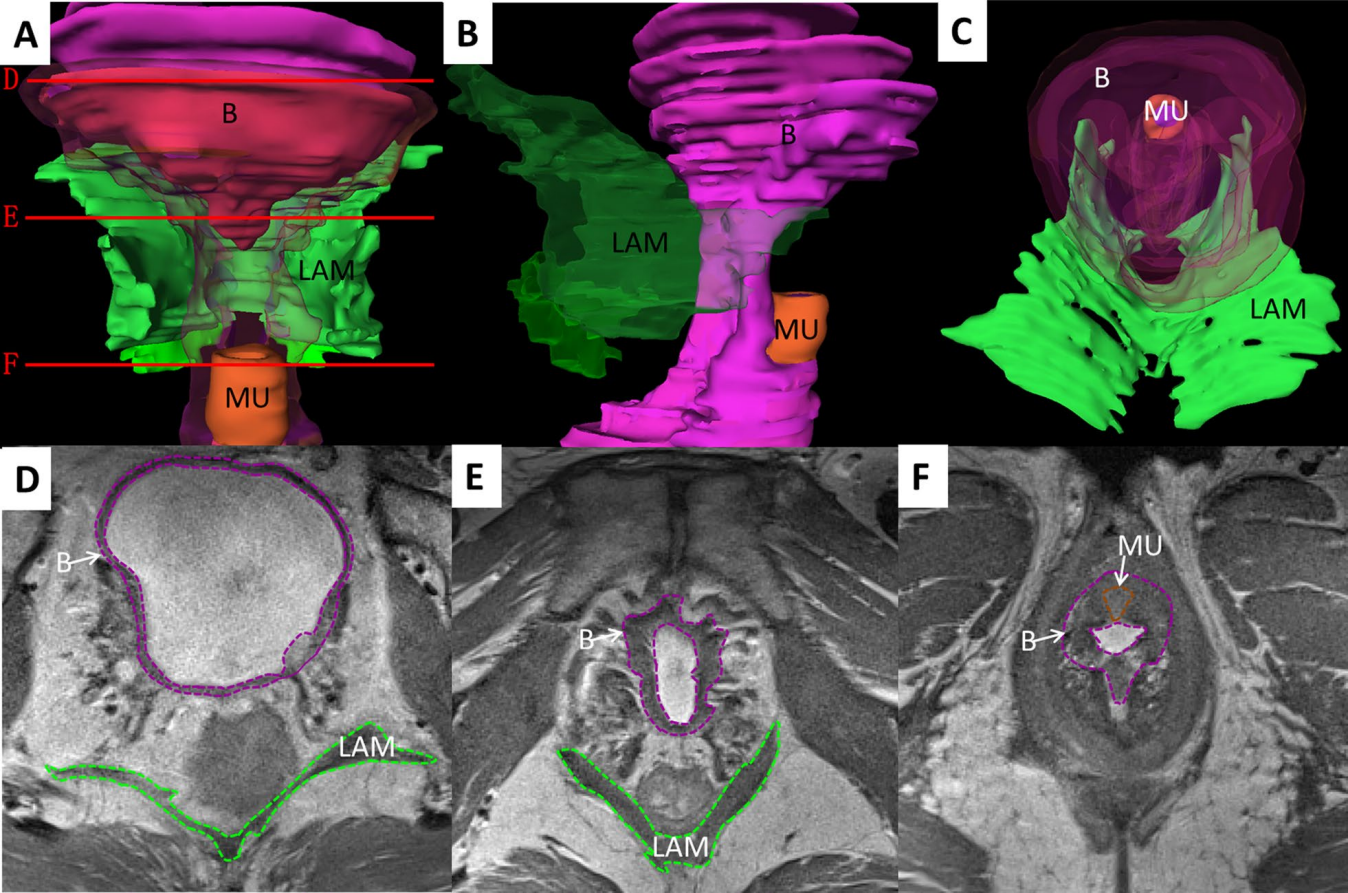
3D models and transverse MRI images of bladder detrusor and urinary continence components of POP patients. **A** Anterior view; **B** Left view; **C** Superior view; **D** Bladder section; **E** Superior urethra section; **F** Inferior urethra section. LAM, levator ani muscle; B, bladder detrusor; MU, main part of urethral sphincter

Additionally, we found significantly widened LAM hiatus of POP patients, prolapsed bladder and uterine components from the orificium vaginae, and certainly damaged LAM. The width of the LAM hiatus was increased in the POP patients as compared to the normal patients, accompanied with a significant difference between the middle and posterior parts of LAM hiatus. The LAM volume in POP patients was notably greater than that in normal patients (Figs. 4, 5).

For the pelvic floor muscle myoelectricity value, the myoelectricity value of the type II muscle (22.9 ± 4.5 uv) and that of type I muscle (19.4 ± 3.8 uv) of OAB patients were remarkably lower than that of the normal group (Table 4).

**Table 4:** Electromyogram of OAB

	Resting values (2–4 uv)	Type II muscle myoelectricity value (35–45 uv)	Type I muscle myoelectricity value (30–40 uv)
OAB	6.6 ± 2.8	22.9 ± 4.5	19.4 ± 3.8

OAB, overactive bladder

Results of Spearman's correlation analysis showed positive correlations of age with the thickness of the bladder detrusor and the width of the posterior part of LAM hiatus and an inverse correlation between the length of the anterior part of LAM hiatus and BMI. The thickness and volume of the bladder detrusor, compressor urethral shared no association with the number of pregnancies, vaginal deliveries, and abortions (Table 5).

**Table 5:** Spearman’s correlation analysis between pelvic floor muscle characters and basic characters in OAB patients

	Age	BMI	Pregnancies	Vaginal deliveries	Abortions
Thickness of bladder detrusor (mm)	0.696*	− 0.216	− 0.015	0.030	− 0.120
Volume of bladder detrusor (mm^3^)	− 0.055	0.123	0.010	− 0.060	0.000
Thickness of compressor urethral (mm)	− 0.055	− 0.333	− 0.163	− 0.030	− 0.180
Length of compressor urethral (mm)	0.091	− 0.141	0.010	0.120	− 0.179
Volume of compressor urethral (mm^3^)	0.055	− 0.137	− 0.056	− 0.179	0.060
Length of the anterior part of LAM hiatus (mm)	0.273	− 0.679*	− 0.326	− 0.179	− 0.239
Length of middle part of LAM hiatus (mm)	0.527	− 0.187	0.398	0.299	0.239
Length of posterior part of LAM hiatus (mm)	0.718*	0.196	0.367	0.060	0.299
Volume of LAM (mm^3^)	− 0.136	− 0.396	− 0.163	− 0.179	0.060

Asterisks indicate the significant correlation at the level of (*) *p* < 0.05 and (**) *p* < 0.01. Correlation coefficient *q* was interpreted as follows: 0.00–0.19 “very weak”, 0.20–0.39 ‘‘weak”, 0.40–0.59 ‘‘moderate”, 0.60–0.79 ‘‘strong” and 0.80–1.0 ‘‘very strong”. BMI, body mass index

**Fig. 6:**
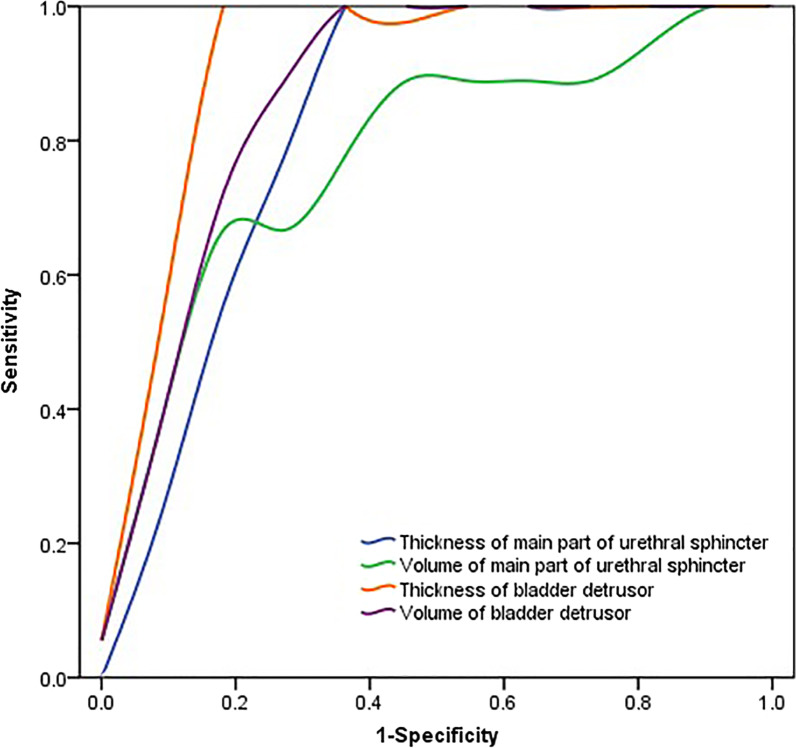
ROC curves of differentiation of pelvic floor muscles’ morphological parameters in OAB group and normal group

We obtained the ROC of the thickness and volume of the main part of urethral sphincter, and the thickness and volume of bladder detrusor, which were 0.823, 0.778, 0.919, and 0.869 separately. We obtained the cutoff value of pelvic floor muscle morphological parameters for OAB group and normal group, The thickness and volume of main part of urethral sphincter were 2.25 mm and 3026.8 mm^3^, The thickness and volume of the bladder detrusor were 2.75 mm and 40,751.5 mm^3^ (Table 6, Fig. 6).

**Table 6:** The AUC, sensitivity, specificity, cutoff value of differentiation of the normal and OAB group

	AUC	Sensitivity (%)	Specificity (%)	Cutoff value
Thickness of main part of urethral sphincter (mm)	0.823	100	63.6	2.25
Volume of main part of urethral sphincter (mm^3^)	0.778	66.7	90.9	3026.8
Thickness of bladder detrusor (mm)	0.919	81.8	100	2.75
Volume of bladder detrusor (mm^3^)	0.869	72.7	100	40,751.5

## Discussion

### Clinical application and advantages of human organ volume measurements

Measurement of human organ volumes has been extensively applied in clinical functional assessment. Muscle volume has been a crucial function assessment indicator of the muscles. To determine the relationship between psoas muscle volume and acute osteoporosis or low bone mass compression fractures in postmenopausal women, Huang et al. have retrospectively analyzed the spinal MRI images and dual-energy X-ray absorptiometry data from postmenopausal women. Their results have uncovered that acute compression fractures are positively correlated with the muscle volume/fat volume ratio and that postmenopausal women with underlying osteoporosis/low bone mass are susceptible to compression fractures due to the small volume of paraspinal muscles. Also, lumbar muscle volume is regarded as the main quantitative and qualitative indicator of its function [5].

Using MRI, de Figueiredo Melke et al. have segmented and reconstructed the total head, upper head, and lower head images of the LPM, and performed 3D quantitative measurement of its volume and insertion patterns, which has shown different volumes of the LPM in the closed and open-mouth positions. Gender may affect muscle volume, especially the upper head, the insertion type of which also affect muscle volume [6]. Jeppe Hvedstrup et al. have measured the volume of the rectus capitis posterior minor muscle through the MRI images of patients with migraine and have revealed no correlations of its volume with migraine, frequency of headache or migraine, and age or BMI, suggesting no pathological structural changes in migraine patients [11].

Therefore, muscle volume is closely linked to its function and disease state, which contributes to accurate diagnosis of diseases, functional assessment, and treatment efficacy evaluation. Through the cutoff value based on the ROC curve, we can diagnose the OAB through directly calculating volume and thickness based on 3D MRI, which is an easy way to diagnose the OAB. In some extent, we may find the OAB through thickness and volume measurement which is higher than the cutoff value of bladder detrusor, and through thickness and volume measurement which is lower than the cutoff value of main part of urethra sphincter.

### The significance of the experimental results

OAB has been previously reported to cause UUI, which is closely relevant to the dysregulated central nervous system. Functional MRI (fMRI) analyses of OAB by Yuko M. Komesu et al. have unraveled the relevance of urinary urgency in OAB patients to limbic cortex (LC) activation, which is likely to be aberrant processing of sensory input in the brain region linked to emotional responses to uncomfortableness [12]. Thus, urinary urgency in OAB is linked to the abnormality in the central nervous system. Matthias Walter et al. observed supraspinal activity responding to bladder distention and cold stimuli, providing a better understanding of the neuropathophysiology of OAB in females. They found impairment in sensory processing and regulation in the OAB patients, which is potentially attributed to insufficient motor activity in the fundamental sensory processing areas, such as the brain insula. Conversely, the cerebellum exhibits considerably enhanced activation of cortical regions, which may support the pelvic floor muscle motor activity to curb urinary incontinence (UI) [13]. However, whether OAB is primarily attributed to abnormal regulation of the central nervous system or impaired anatomical components of urination and UC remains unclear.

Our preliminary study and Christian Wallner's study have shown that the bladder detrusor is a key urination anatomical component and that the LAM and USC such as the main part of urethral sphincter, the compressor urethral, and the urethrovaginal sphincter are important UC anatomical components. Based on the previous anatomical research results, this study additionally unraveled the increased volume of urination anatomical component detrusor in the OAB patients versus the healthy volunteers. This is attributable to long-term muscle tension or contraction of the bladder and its hypercontraction, leading to significantly thickened bladder detrusor and increased volume. The increase of the muscle is more likely to induce a long-term continuous urge to urinate, resulting in UI.

However, the general topology and volume of the LAM showed no noteworthy difference between the OAB patients and the volunteers. Also, no obvious structural damage and changes of the LAM were found in OAB patients, accompanied by no LAM atrophy and volume reduction. From the perspectives of the anterior, middle, and posterior pelvic widths of the LAM hiatus, the hiatus of anterior pelvis was generally wide while the middle and posterior pelvises were almost unaffected, indicating a certain morphological change of the anterior pelvic part of the LAM. Due to long-term UI, the anterior pelvic part of the LAM is loose, but it is far from functional disorders of the pelvic floor such as POP. The LAM is significantly damaged in the patients with long-term prolapse, characterized by relatively large structural damage, and obvious compensatory hypertrophy of the LAM, significantly increased volume, and obvious widen LAM hiatus. MRI study of DeLancey et al. have illustrated that vaginal delivery may cause LAM damage [14], whereas, their research is only limited to the two-dimensional MRI images but lacks either 3D localization or 3D quantification. DeLancey et al. have shown noticeable LAM injury, a significant increase in width of the LAM hiatus, and notably weakened muscle strength in the POP patients as compared to conventional normal volunteers by MRI [15]. DeLancey et al. have measured the volume of the puborectalis muscle in healthy volunteers and POP patients using MRI and 3D reconstruction techniques, demonstrating no notable difference between groups and no puborectalis muscle defect. As illustrated in their article, although there is little difference in the puborectalis muscle between the two groups, the proportion of thickened puborectalis in the POP patients was much higher than in the volunteers. Birth trauma is an important cause of LAM damage, a crucial inducer of POP [14, 16].

We also found weakened main part of urethral sphincter and the compressor urethral in the USC of OAB patients than healthy volunteers except that it is difficult to identify and segment the urethrovaginal sphincter in MRI images [1, 2]. The volume and thickness were markedly smaller in OAB patients than in healthy volunteers, which led to weakened continence muscles and easy leakage of urine, causing or aggravating UUI. Our findings are in agreement with the results of Wu et al. and Wallner et al. [1, 2]. Therefore, the volume of the USC can serve as a quantitative parameter for female UC. Additionally, the results of this study also validated prominently lower pelvic floor muscle strength of OAB patients than the normal values.

MRI measurement by Cevdet Adıgüzel et al. has elucidated no significant difference regarding the thicknesses of the left and right uterosacral ligament (USL) between the OAB patients and the control group, indicative of no relation between the condition of OAB and the USL [17]. Therefore, the occurrence and symptoms of OAB are mainly related to the female urination and continence muscles, instead of the uterine ligaments.

These results offered the first quantitative evidence for pelvic floor imaging studies. Our data of Spearman correlation analysis further unveiled that OAB was strongly linked to age, BMI, pregnancies, and vaginal deliveries, rather than abortions. The incidence of OAB increases with the age, which correlates with the decline of estrogen and autonomic disorders [18]. More pregnancies correlate with a longer cumulative time of compression over the pelvic floor, which is easier to cause disorders in the autonomic nervous system and resultant urodynamic disorders, increased frequency of urination, as well as tore and damaged pelvic floor muscles and widened LAM hiatus, resulting in a risk of the organs prolapse. Besides, the LAM hiatus is easily damaged following vaginal delivery, which will widen the LAM hiatus and lead to the LAM fiber destruction, ultimately contributing to UI. Also, obese patients with higher BMI are at a higher risk of OAB, and more severe symptoms are related to high incidence of POP. As the bladder is compressed by the larger body weight, the prolapse and UI may be aggravated. Obesity (BMI greater than 30 kg m^2^) and overweight (BMI greater than 25–30 kg m^2^) are associated with increased prevalence of pelvic floor disorders including UI and OAB. OAB can be triggered by several obesity-related disorders, such as mechanical compression over the bladder caused by weight gain, type 2 diabetes, or metabolic syndrome, the latter two of which elicit an underlying metabolic defect during the occurrence of OAB [2, 19]. Moreover, excessive fat in obese patients will destroy the dense junctions between the pelvic floor muscles and alter the dense connective tissues with tight junctions into fat with loose junctions, resulting in pelvic floor relaxation and UI [20].

## Conclusions

We used MRI 3D reconstruction in the normal volunteers, the OAB and the POP patients. In OAB patients, the bladder detrusor has long-term tension and contraction, which thickened muscle and increased volume, and aggravate urination, while the compressor urethral and main part of urethral sphincter are weaker and the anterior part of LAM hiatus is relaxed, easily resulting in leakage of urine and ultimately incontinence. The MRI 3D reconstruction and measurement can help to evaluate pelvic floor urination and continence function, and accurately diagnose.

## Limitation

Our study was limited by a small number of subjects, because we should enroll the patients in the hospital to get thin-sectional high-resolution MRI images with special MRI sequence. In the future study, we should expand our sample size.

## Data Availability

The datasets used or analysed during the current study are available from the corresponding author on reasonable request.

## Abbreviations

OAB: Overactive bladder
UUI: Urge urinary incontinence
BMI: Body mass index
UC: Urinary continence
USC: Urethral sphincter complex
LAM: Levator ani muscle
3D: Three-dimensional
MRI: Magnetic resonance imaging
LPM: Lateral pterygoid muscle
POP: Pelvic organ prolapse
fMRI: Functional MRI
LC: Limbic cortex
UI: Urinary incontinence
USL: Uterosacral ligament
CVH: Chinese Visible Human
B: Bladder detrusor
MU: Main part of urethral sphincter
CU: Compressor urethrae
